# CCS-mediated mechanistic link between gestational diabetes mellitus and carpal tunnel syndrome: a multi-omics MR framework

**DOI:** 10.3389/fimmu.2026.1766134

**Published:** 2026-03-11

**Authors:** Rui Chen, Yu Zhang, Xiangbo Meng, Xingyu Ren, Teng Lv, Yihua Sun, Tao Chen

**Affiliations:** 1 Department of Reproductive Medicine, The Affiliated Hospital of Qingdao University, Qingdao, Shandong, China; 2 Department of Obstetrics and Gynaecology, The University of Qingdao, Qingdao, China; 3 Department of Gynaecology, The Affiliated Hospital of Qingdao University, Qingdao, Shandong, China; 4 Department of Ultrasound, The Affiliated Hospital of Qingdao University, Qingdao, Shandong, China; 5 Department of Radiology, The University of Qingdao, Qingdao, China

**Keywords:** Bayesian colocalization, carpal tunnel syndrome, gestational diabetes mellitus, Mendelian randomization, phenome-wide association study

## Abstract

**Background:**

Carpal tunnel syndrome (CTS) is a common condition in pregnancy, yet reliable tools for identifying women at high risk—particularly those with gestational diabetes mellitus (GDM)—remain lacking. Although GDM shares metabolic features with type 2 diabetes, a recognised CTS risk factor, whether GDM itself causally increases CTS risk and through which molecular pathways has not been established.

**Methods:**

We used linkage disequilibrium score regression and two-sample Mendelian randomization across FinnGen and UK Biobank to evaluate the genetic correlation and causal effect of GDM on CTS. To identify molecular mediators, we integrated CTS GWAS with whole-blood cis-eQTLs and plasma protein QTLs using summary-data MR and Bayesian colocalization. We then characterised trait specificity through phenome-wide MR and quantified mediation effects through two-step MR. Bulk RNA-seq of CTS tissue, single-cell RNA-seq of placental cell from women with and without GDM, murine histology and immunofluorescence, and molecular docking were used to delineate downstream mechanisms and therapeutic potential.

**Results:**

GDM and CTS showed significant genetic correlation (rg = 0.219). Genetic liability to GDM causally increased CTS risk across discovery, replication, and female-only models, independent of other metabolic or pregnancy-related traits. Multi-omics integration identified CCS as the only gene supported at both eQTL and pQTL levels and revealed its strongest and most specific causal association with CTS. Mediation MR demonstrated that circulating CCS accounts for a substantial proportion of the GDM–CTS effect. Transcriptomic, single-cell, and animal analyses confirmed a CCS-high, inflamed, and collagen-rich microenvironment in CTS, whereas docking analyses indicated that CCS-centred pathways are pharmacologically tractable.

**Conclusion:**

GDM exerts a causal effect on CTS, largely mediated through CCS-driven oxidative, immune, and fibrotic pathways. CCS emerges as a promising biomarker for risk stratification and a potential therapeutic target. These findings provide a mechanistic foundation for early CTS surveillance and personalised management in women with GDM, addressing an important unmet clinical need in perinatal care.

## Introduction

Carpal tunnel syndrome (CTS) is the most common entrapment neuropathy, caused by compression of the median nerve within the rigid osteofibrous tunnel at the wrist (1). Clinically, CTS presents with numbness, burning pain, and paraesthesia in digits 1-3, often radiating proximally to the forearm and shoulder (1). In advanced disease, patients may develop marked motor deficits and cutaneous changes, including erythema, oedema, and blistering, with a substantial impact on hand function and quality of life (2). Diabetes mellitus, particularly type 2 diabetes (T2D), is a well-established determinant of CTS, likely through converging mechanisms of advanced glycation, microangiopathy, and chronic low-grade inflammation (3–5). Pregnancy itself is also an independent risk state: Up to 62% of women in the third trimester develop CTS-like symptoms, driven by fluid retention, increased intracarpal pressure, and hormonally mediated ligamentous laxity (6).

Gestational diabetes mellitus (GDM), defined as glucose intolerance first recognised during pregnancy in women without pre-existing diabetes (7), sits at the intersection of these two risk domains. GDM combines metabolic disturbances reminiscent of T2D, such as insulin resistance and chronic hyperglycaemia, with the unique haemodynamic, hormonal, and volume changes of pregnancy. This constellation plausibly amplifies median nerve vulnerability through oedema, microvascular dysfunction, and oxidative stress. However, the study linking GDM to CTS has been observational (8)and, therefore, remain susceptible to confounding, reverse causation, and imprecise temporal relationships. Whether GDM itself causally increases CTS risk or co-occurs with other metabolic and obstetric factors remains unclear.

From a pathophysiological perspective, GDM arises when pregnancy-induced insulin resistance is not adequately compensated by β-cell adaptation, resulting in relative insulin deficiency and hyperglycaemia (9). Women with a history of GDM are at substantially elevated risk of progressing to T2D, underlining the shared metabolic substrate between these conditions (10). Given the established association between T2D and CTS (11) and emerging reports that GDM is associated with higher rates of CTS symptoms and surgery (12), there is a strong rationale for rigorously testing whether GDM is an actual causal driver of CTS rather than a mere correlate of broader metabolic dysfunction. Clarifying this relationship is crucial for risk stratification, guideline development, and the design of targeted preventive interventions in pregnant women.

Mendelian randomization (MR) provides a robust framework to address these questions by using germline genetic variants, typically single-nucleotide polymorphisms (SNPs), as instrumental variables for modifiable exposures (13). Because these variants are randomly allocated at conception and fixed throughout life, MR analyses are less prone to confounding and reverse causation than classical observational studies. MR has already clarified several ambiguous links between cardiometabolic traits and neuropathic outcomes, illustrating its utility in dissecting complex metabolic–neurological interactions. At the same time, advances in multi-omics resources, including blood-derived expression quantitative trait loci (eQTLs) and protein quantitative trait loci (pQTLs), now allow integration of MR with gene- and protein-level mapping to pinpoint putative effector molecules and pathways.

In this study, we applied an integrated, multi-stage MR strategy-encompassing bidirectional, multivariable, and mediation MR to interrogate the causal relationship between GDM and CTS. We combined large-scale GWAS data with blood-derived cis-eQTL and pQTL datasets. We used summary-data-based MR (SMR) plus Bayesian colocalization (COLOC) to prioritise genes and proteins whose regulation is plausibly on the causal pathway. We then extended these findings using bulk RNA sequencing of carpal tunnel tissue, single-cell RNA sequencing of placental cells from women with and without GDM, and histopathological assessment in a GDM mouse model, to delineate the cellular and tissue microenvironment associated with CTS. Finally, we evaluated the druggability of key targets using structure-based molecular docking.

Our aims were threefold: first, to determine whether GDM causally increases CTS risk in women; second, to define the molecular mechanisms underlying this association, with particular attention to metabolic, oxidative-stress, immune, and extracellular matrix pathways; and third, to identify tractable gene and protein targets that could inform precision prevention or mechanism-based therapy in metabolically vulnerable populations. By integrating genetic epidemiology with multi-omics and experimental data, we aimed to connect clinical observations with mechanistic insights to provide a rational foundation for early surveillance and intervention of CTS in women affected by gestational diabetes mellitus (GDM).

## Materials and methods

### 2.1 Integrative omics and causal inference analysis

At the genomic level, summary-data-based Mendelian randomization (SMR) analysis was performed utilizing large-scale GWAS summary statistics from the FinnGen consortium and the UK Biobank. This analysis aimed to identify genes that were putatively causally associated with CTS. To validate these findings at the proteomic level, we subsequently conducted MR analysis using protein quantitative trait loci (pQTL) data derived from circulating protein measurements.

### 2.2 Genetic colocalization analysis

To strengthen the causality of identified gene-disease associations and distinguish shared genetic aetiology from linkage, we performed Bayesian colocalization analysis. Specifically, this analysis was applied to the prioritised candidate gene loci derived from the SMR analysis. For each locus, we assessed whether the genetic variants influencing gene expression (eQTLs) or protein abundance (pQTLs) were shared with those conferring risk for carpal tunnel syndrome (CTS). The analysis utilised the same GWAS summary statistics from the FinnGen and UK Biobank cohorts, alongside matched QTL data sources. A posterior probability of colocalization (PP.H4) >0.8 was used as the primary threshold to define a high-confidence shared causal variant, thereby pinpointing specific genomic loci where molecular QTL and disease GWAS signals are likely driven by the same underlying genetic mechanism.

### 2.3 Bulk RNA sequencing and pathway analysis

Batch RNA-seq data from CTS and healthy carpal tunnel tissues in GSE108023 were analysed using DESeq2 (14, 15). Differentially expressed genes were defined as FDR < 0.05 and |log_2_FC| > 1. Functional enrichment using KEGG and Gene Ontology was performed with clusterProfiler to identify immune-activation and extracellular-matrix (ECM) remodelling pathways (16–18).

### 2.4 MR-PheWAS analysis

To assess the broad clinical relevance and phenotypic specificity of CCS in human populations, we conducted a phenome-wide Mendelian Randomization analysis. Genetic instruments for CCS were constructed using independent cis-pQTLs (independence threshold: LD r^2^ = 0.1, kb = 10,000) identified from large-scale plasma proteome studies (https://www.decode.com/summarydata/) (19). We then systematically evaluated the causal associations between genetically predicted CCS levels and a wide array of phenotypes using GWAS summary statistics from the UK Biobank and FinnGen consortium. The significance of associations was determined after multiple testing correction using a false discovery rate threshold (FDR < 0.05).

### 2.5 Single-cell RNA-seq analysis

#### 2.5.1 Preprocessing

Placental cell scRNA-seq data were obtained from GSE173193 from two GDM patients and two normoglycated controls and processed using Seurat (v5) (20, 21). To remove cells with low RNA counts, low feature counts, or high mitochondrial gene percentages, we initially filtered to retain genes expressed in at least three cells and cells with at least 200 detected genes. Cells were filtered to retain those with 200-6,000 detected genes and <15% mitochondrial content. Data were normalised using LogNormalize with a scale factor of 10,000 (22).

#### 2.5.2 Clustering and annotation

For each sample, 2,000 highly variable features were identified using variance stabilizing transformation (VST). Following data integration, all genes underwent z-score standardization (23). Highly variable genes were identified for PCA. To correct for sample-specific batch effects, the Harmony algorithm was applied using “sample” as the batch variable (24). UMAP was applied for dimensionality reduction and visualizations, followed by clustering (FindClusters) (25). We used a manual annotation method for cell annotation and constructed a reference panel of 17 major placental cell types based on literature and databases (CellMarker, PanglaoDB) (26). For each cluster, we calculated a matching score by quantifying the overlap between the cluster’s top 50 marker genes (identified by Wilcoxon test) and each reference cell type’s marker set. The cell type with the highest score was assigned as the predicted identity. Marker expression levels and detection rates were computed using Seurat’s FetchData function and visualised on UMAP embeddings to validate biological coherence.

#### 2.5.3 Gene profiling

Differential expression analysis between GDM and control groups was conducted at two levels: global comparison across all cells and cell type-specific comparisons within each cluster. Statistical tests were performed using the Wilcoxon rank-sum test. Multiple testing correction was performed using the Bonferroni method. Genes with |log2FC| > 0.5 and adjusted p-value < 0.05 were considered significantly differentially expressed (14). CCS, NEK7, and other MR-prioritised genes were analysed across clusters using UMAP feature plots and violin plots to identify CCS-high interferon-responding populations enriched in GDM.

#### 2.5.4 Pathway and communication analysis

GSVA quantified pathway activities using MSigDB hallmark gene sets (27, 28). CellChat and CellPhoneDB inferred ligand–receptor interactions, focusing on signalling from interferon-responding cells to epithelial-barrier cells, particularly ECM-related pathways (29, 30).

#### 2.5.5 Trajectory inference analysis

To investigate developmental trajectories and GDM-induced lineage transitions, pseudotime analysis was performed on selected cell populations (clusters 2, 4, 8, 11, and 15). The steps are to convert the Seurat object into a SingleCellExperiment object and analysed using the Slingshot algorithm, infer cell developmental trajectories based on UMAP dimensionality reduction space, and designate cluster 4 as the starting point (31). Calculate cell pseudotime and identify developmental trajectory branches.

#### 2.5.6 Statistical analysis and visualization

All statistical analyses were performed using R (v4.5.1). Primary packages included Seurat (v5) for single-cell analysis, dplyr for data manipulation, ggplot2 for visualization, patchwork for plot assembly, harmony for batch correction, and slingshot for trajectory inference. Visualizations included violin plots, UMAP plots, heatmaps, dot plots, volcano plots, density plots, and box plots. All figures were generated in PDF vector format at 300 DPI resolution for publication quality.

### 2.6 Animal experiments, immunohistochemistry, and immunofluorescence

We created a model of gestational diabetes mellitus (GDM) in mice by feeding them a high-fat diet along with a low dose of a drug called streptozotocin. A total of 30 female SPF-grade C57BL/6 mice (3 weeks old) with body weights controlled within a specific range (18–22 g) were selected. Before mating, the female mice were fed a high-sugar, high-fat diet (HFD) (typically containing a high proportion of fat and sucrose, with 50% fat and 15% sucrose) for 4 weeks to induce insulin resistance and lay the foundation for the development of gestational diabetes mellitus. There were 15 male mice introduced at a female-to-male ratio of 2:1 for overnight co-housing. Vaginal plugs were checked the next day, and the day a plug was detected was designated as gestation day 0 (GD0). After confirming pregnancy, the GDM group continued to receive the high-sugar, high-fat diet until the experimental endpoint (e.g., gestation days 18–20). On gestation day 1 (GD1), blood was collected from the tail vein of pregnant mice to measure initial fasting blood glucose (FBG). Pregnant mice with FBG ≤6.1 mmol/L were selected and subsequently divided into a normal control group (control, n = 9) and a modelling group (n = 11, FBG ≥ 7.0 mmol/L). Blood glucose monitoring: FBG was measured during gestation (e.g., GD7, GD14, and GD18). If FBG was significantly higher than that of the normal pregnancy group (e.g., ≥7.0 mmol/L), it indicated abnormal glucose metabolism. Ultimately, six mice in the GDM group had abnormal FBG in all four measurements, and seven in the control group had normal FBG in all four measurements.

Wrist-canal tissues (experimental group: five GDM mice; control group: five healthy pregnant mice) were collected in late gestation, rapidly embedded in OCT, and processed as cryosections for downstream analyses. For histological evaluation, frozen sections were fixed in 4% paraformaldehyde and stained with haematoxylin–eosin and Masson’s trichrome to assess inflammatory infiltration and extracellular matrix (ECM) thickening. Images were acquired under standard light microscopy and quantified using ImageJ. For immunofluorescence, cryosections were permeabilised with 0.1% Triton X-100 and incubated with primary antibodies against CCS (rat monoclonal, Alexa Fluor 647 conjugate) and ECM markers, followed by nuclear counterstaining with DAPI. Confocal images were obtained using a Leica TCS SP8 system. To verify interferon-γ detection, mouse splenocytes were fixed, permeabilised, and stained with anti-interferon-γ (ab324874, 1:250) and Alexa Fluor 488-conjugated secondary antibody (ab150081, 1:1,000). Secondary-only controls were included in all assays.

We used a method called “quantitative immunofluorescence” (Fiji/ImageJ; background-corrected mean fluorescence). Red-channel images of CCS staining were captured using the same microscope settings for each group (exposure/laser power, gain, and offset were kept constant) and stored at the original bit depth for analysis. The images were opened in Fiji/ImageJ, and the red channel was analysed as a black-and-white image (channels were split when needed). Single-cell ROIs were clearly defined within each field and stored in the ROI Manager. We estimated the background from a cell-free area in the same field to get the background mean grey value (Mean_bg). For each cell ROI, the mean grey value (Mean_cell) was measured, and the background-corrected mean fluorescence was calculated as Mean_bg-corrected = Mean_cell − Mean_bg. Then, we put all the measurements together at the predefined experimental unit (wrist-canal tissues) to compare them statistically.

### 2.7 Western blot and qPCR

Western blotting was performed according the standard protocol, including total cellular protein extraction, quantification, electrophoresis, and transfer to membrane. This was followed by blocking, sequential incubation with primary antibodies (CCS: Cat No. 68341-1-Ig; GAPDH: Cat No. 60004-1-Ig) and secondary antibodies. GAPDH served as loading control.

For qPCR, RNA was extracted using TRIzol. A NanoDrop 2000 spectrophotometer was utilised to measure the concentration and purity of RNA. Equal amounts of RNA were reverse-transcribed into cDNA following the manufacturer’s instructions. Using SYBR Green dye, qPCR was performed on a real-time PCR system. cDNA, gene-specific primers, and SYBR Green master were mixed in each 20-µL reaction. The reaction conditions were 95 °C for 30 s and then 40 cycles of 95 °C for 5 s and 60 °C for 30 s. Each sample was analysed in triplicate, and the 2^−ΔΔCt method was performed to calculate gene expression levels, with GAPDH as internal control. Primer sequences are provided in **Supplementary Table S1**.

### 2.8 Molecular docking

Candidate proteins (CCS, NEK7, PTPN9, ASPN, MST1, and ITGA2) were selected based on convergent MR and colocalization evidence. Ligand structures for Amiloride (PubChem CID:16231) and Tinlarebant (PubChem CID:92044505) were downloaded from PubChem and converted to mol2 format using Open Babel. Protein structures were retrieved from the AlphaFold. Protein Structure Database, stripped of water and co-crystallised ligands in PyMOL, and processed in AutoDockTools for hydrogen addition and charge assignment. Ligands and receptors were converted to PDBQT format. Docking was performed using AutoDock Vina (exhaustiveness = 4). Binding energies < −5.0 kcal/mol were considered indicative of favourable interaction. Top docking poses were visualised in PyMOL. Import the docking results into PyMOL, create overall and zoomed-in images by displaying the hydrogen bonds and the amino acid residues connecting the receptor and ligand, and show the names of the amino acid residues (32).

### 2.9 Study design

**Figure 1:**
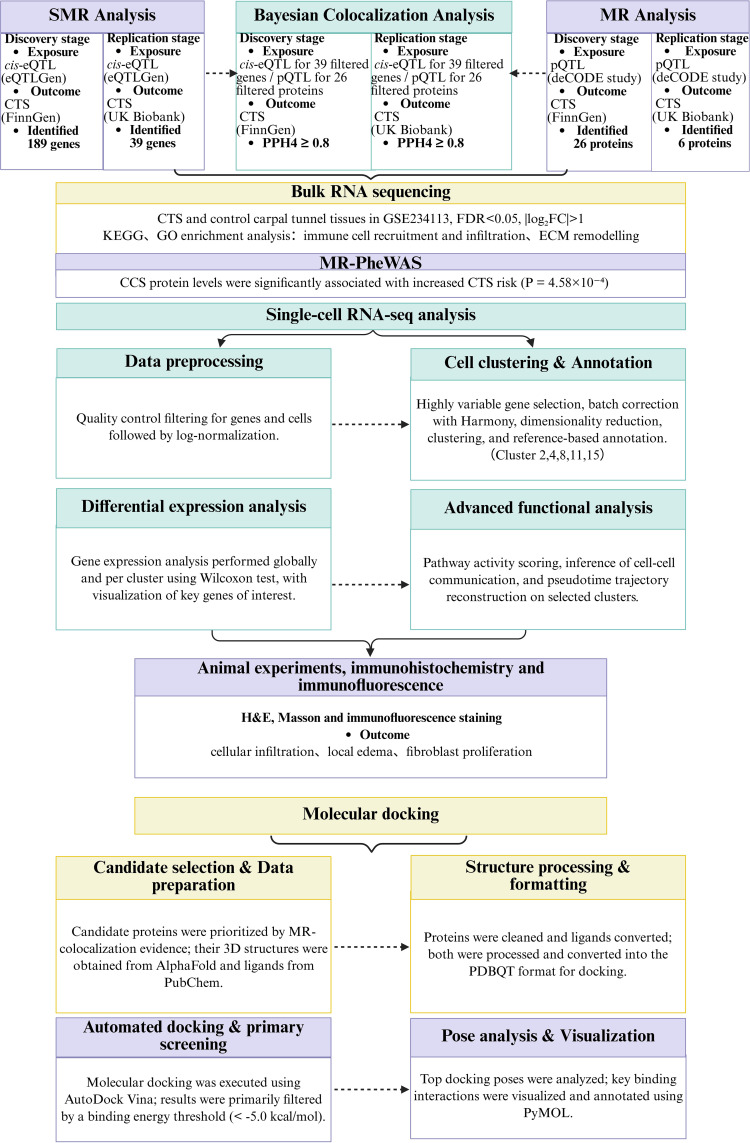
Overview of the study design. This figure illustrates the sequential analytic framework used to identify causal pathways linking GDM to CTS: (i) SMR integrating whole-blood cis-eQTLs with two independent CTS GWAS datasets; (ii) pQTL-based MR to evaluate circulating proteins; (iii) Bayesian colocalization to identify shared causal variants; (iv) Bulk RNA-seq of CTS tissue to detect transcriptional dysregulation in colocalised genes; (v) Single-cell RNA-seq of placental cell to map CCS-related immune alterations in GDM; (vi) Molecular docking to assess druggable interactions; and (vii) Histology, immunofluorescence, qPCR, and western blot validation in the GDM mouse model. GDM, gestational diabetes mellitus; CTS, carpal tunnel syndrome; SMR, summary-data Mendelian randomization.

We used a structured, multi-stage analytical framework (**Figure 1**). First, linkage disequilibrium score regression (LDSC) was applied to quantify the genetic correlation between gestational diabetes mellitus (GDM) and carpal tunnel syndrome (CTS). We then performed bidirectional Mendelian randomization (MR) to test causality between GDM and CTS, using FinnGen and UK Biobank as independent discovery and replication datasets. To assess the independence and robustness of the GDM–CTS association, we carried out univariable (UVMR) and multivariable MR (MVMR) analyses, adjusting for correlated traits from FinnGen, including diabetes-related traits (other disorders of glucose regulation and pancreatic internal secretion [ODGRPIS], type 1 diabetes [T1D], type 2 diabetes [T2D], insulin-treated diabetes [DM-Insulin]), hypertension-related traits (hypertension [HTN], gestational hypertension [GHTN], pre-existing hypertension complicating pregnancy [PreHTN Preg]), lipid-related traits (hyperlipidaemia [HL], mixed hyperlipidaemia [MHL]), and pregnancy-related traits (single spontaneous delivery [SSD], other maternal disorders predominantly related to pregnancy [MatDis Other]).

To map gene- and protein-level mediators of CTS, we next performed summary-data-based MR (SMR) by integrating whole-blood cis-eQTL data from the eQTLGen Consortium with CTS GWAS summary statistics from FinnGen (discovery) and UK Biobank (replication). Genes passing SMR and heterogeneity filtering were further evaluated by Bayesian colocalization (COLOC), and high-confidence causal genes were defined using a combined posterior probability threshold (PPH4 ≥ 0.80). In parallel, we analysed protein quantitative trait loci (pQTL) from the deCODE proteomics study (4,907 circulating proteins;https://www.decode.com/summarydata/) using MR and COLOC to identify CTS-related proteins, with particular focus on CCS, HEXIM2, HEXIM1, CPZ, and PTPN9. For CCS, we examined colocalization between eQTL/pQTL signals and CTS GWAS peaks at the locus level to confirm that regulation of CCS and CTS risk was driven by shared variants. Downstream pathway analysis of differentially expressed genes was performed using KEGG and Gene Ontology (GO), and histological validation with H&E and Masson staining in GDM and control mice was used to compare inflammatory cell infiltration, oedema, and fibroblast proliferation.

To characterise the broader clinical profile of CCS, we performed a phenome-wide MR (MR-PheWAS) across a large panel of FinnGen traits and then applied two-step mediation MR to formally test whether circulating CCS levels mediate the causal effect of GDM on CTS by partitioning the total effect into CCS-mediated (indirect) and non-mediated (direct) components. On the cellular level, we used CCS expression patterns in single-cell RNA-seq data to identify and cluster high-expressing populations, focusing on clusters 2, 4, 8, 11, and 15. Differentially expressed genes within the key cluster (cluster 15) were analysed by pathway enrichment to define functional programmes linked to CCS. Finally, we assessed the druggability of candidate causal genes emerging from SMR and COLOC by molecular docking of two small molecules against CCS and other targets and evaluated binding affinity and interaction profiles at the protein–ligand interface. All reporting strictly adhered to the STROBE-MR guidelines (33), with comprehensive methodological details and genetic instrument descriptions available in **Supplementary Table S2**.

### 2.10 Data sources

Data sources for each phenotype are detailed in **Supplementary Table S3**. Summary genetic data pertaining to GDM were acquired from the FinnGen cohort due to its high genetic homogeneity and rich, registry-based phenotyping, comprising 18,581 cases and 263,483 controls, alongside sex-stratified GDM data from the UK Biobank datasets as replication, which include 691 cases and 5,428 controls. The UK Biobank’s larger, more genetically diverse population enables the assessment of the robustness and generalizability of the discovered associations across a broader demographic context. Additionally, the genome-wide association study (GWAS) analysis for common traits and symptoms (CTS) was extracted from the UK Biobank consortium, involving 8,289 cases and 454,721 controls. For replication purposes, the GWAS data for CTS was further sourced from the FinnGen consortium, which encompasses 31,181 cases and 435,371 controls. In the UVMR and MVMR analyses, the diabetes-related traits, hypertension-related traits, lipid-related traits, and pregnancy-related traits were abstracted from the FinnGen cohort, the CTS from the UK Biobank consortium. Moreover, we employed SMR analysis utilising whole blood cis-eQTLs (the eQTLGen Consortium) and two independent CTS GWAS meta-analyses (discovery: FinnGen; replication: UK Biobank) to investigate the causal effects of gene expression on susceptibility to CTS (34). Furthermore, genes identified as significant in SMR were further validated through COLOC analysis in the discovery stage (CTS: FinnGen) and replication stage (CTS: UK Biobank). Additionally, we extended our analysis to include pQTL data from the deCODE proteomics study, which measured 4,907 circulating proteins. Notably, the genetic correlation between GDM (FinnGen) and CTS (UK Biobank) was additionally assessed via linkage disequilibrium score regression (LDSC).

The GWAS data of the diseases mentioned above were collected according to the World Health Organisation (WHO) criteria. Furthermore, CTS cases were determined by referencing the ICD-10 code G56.0 within diagnostic records.

The bulk RNA-seq data used in this analysis were obtained from the GEO database (accession number: GSE108023). The GSE108023 data were obtained from tenosynovium samples collected during carpal tunnel decompression in 41 patients with CTS and from healthy skin samples collected from six healthy subjects for RNA sequencing. Furthermore, placental single-cell RNA-seq data used in this analysis were obtained from the GEO database (accession number: GSE173193). Single-cell RNA sequencing technology was used in the GSE173193 dataset. Placental tissues of two patients with gestational diabetes mellitus (GDM) and two normal pregnant patients were recruited from Changzhou Maternal and Child Health Care Hospital, affiliated with Nanjing Medical University.

### 2.11 SNP selection

To utilise genetic variation in assessing the causal association between exposure and outcome, it must meet the three core assumptions for instrumental variables (IVs) (35): (i) Relevance Assumption—IVs were strongly related to the exposure at a genome-wide significant level (p < 5×10^-8^). The mean F-statistic (F = (Beta/Se) ²) for each SNP was calculated (36, 37). An F-statistic weaker than 10 indicated a weak association between SNP and exposure, which should be excluded. (ii) Independence Assumption—the genetic variants had no association with any confounding factors that could affect the exposure and outcome. Due to the linkage disequilibrium (LD) structure in the genome, significant associations between genetic variants and traits were identified at a P < 5 × 10^−8^ threshold, r² < 0.001, and a 10,000-kb window size (38). We utilised the PhenoScanner tool to determine whether the IVs were significantly correlated with other risk factors for CTS (39, 40). (iii) Exclusion Restriction Assumption—the genetic variants affected CTS only through exposures. SNPs that failed the Steiger test of directionality (one-sided P ≥ 0.05) were removed.

### 2.12 Statistical analyses

We estimated the genetic correlation (r_g_) between GDM and CTS using LDSC (41). GWAS summary statistics were filtered, excluding non-SNP variants (e.g., indels), strand-ambiguous SNPs, duplicate SNPs, and SNPs with minor allele frequency (MAF) < 0.01. The genetic covariance was estimated by regressing the product of the z-scores from GDM and CTS for each variant against its LD score. LDSC quantifies the contribution of true polygenic signal versus bias to test statistic inflation by examining the association between test statistics and linkage disequilibrium (42). Additionally, the bidirectional MR was utilised to examine the genetic relationship between GDM and CTS risk. Specifically, inverse variance weighted (IVW) was used as the primary approach for MR (43, 44). The complementary methods included the robust adjusted profile score (RAPS) (45), constrained maximum likelihood (CML) (46), debiased inverse-variance weighted method (dIVW) (47), and Bayesian weighted Mendelian randomization (BWMR) (48). The Wald ratio method was employed to evaluate associations for genetic variants comprising a single SNP. We then performed sensitivity analyses to test the independence and exclusion assumptions. In detail, we employed MR-Egger regression methods to identify potential pleiotropy (49, 50). Then, Cochran’s Q test was used to determine heterogeneity among IVs (44, 51). A leave-one-out analysis was also conducted to identify single SNPs that could potentially affect the results. For UVMR, we used the IVW method, and for MVMR we primarily employed multivariable IVW (MV-IVW) (52, 53). Furthermore, integration of whole blood cis-eQTLs with the two independent CTS GWAS meta-analyses was performed using SMR and COLOC analyses (54). To further elucidate molecular mechanisms, significant protein-level associations were similarly assessed via MR and COLOC. Owing to consistent causal evidence for CCS across transcriptomic and proteomic layers, Colocalization analysis was performed to assess genetic colocalization; pathway enrichment analysis was conducted using the GO and KEGG databases; and histopathological evaluation of wrist tunnel tissue was carried out via HE and Masson staining. An MR-PheWAS was performed to evaluate its trait specificity and clinical relevance (55), and colocalization analysis was performed to assess genetic colocalization. A two-step mediation MR analysis tested whether CCS protein levels mediate the GDM–CTS causal relationship (56). Finally, we assessed the druggability of integrated SMR/COLOC-identified causal genes to prioritise therapeutic targets. We conducted all analyses in RStudio 4.3.2.

## 3 Results

### 3.1 Blood-derived gene and protein regulation contributes causally to carpal tunnel syndrome susceptibility

**Figure 2:**
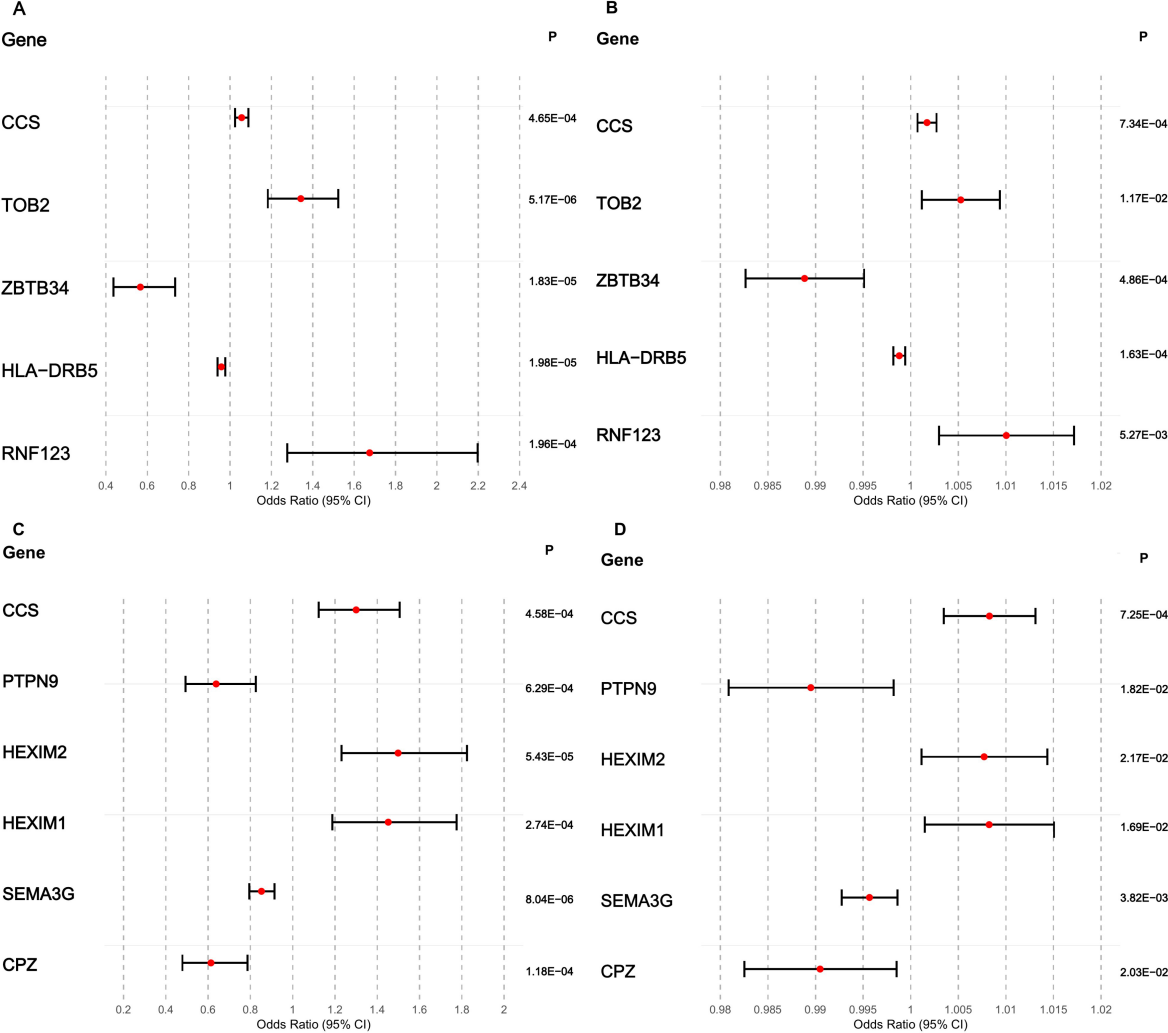
Causal effects from a few of eQTL-SMR and pQTL-MR analyses (the details were provided in **Supplementary Figure S1**). **(A)** SMR estimates for a few of cis-eQTLs associated with CTS risk in FinnGen. **(B)** SMR estimates for a few of cis-eQTLs associated with CTS risk in UK Biobank. **(C)** MR estimates for a few of circulating proteins (pQTLs) influencing CTS risk in FinnGen. **(D)** MR estimates for a few of circulating proteins influencing CTS risk in UK Biobank. Red dots indicate effect estimates, and horizontal lines represent 95% confidence intervals.

We first investigated whether whole-blood gene expression influences CTS risk by applying summary-data Mendelian randomization (SMR) to integrate cis-eQTL data with CTS GWAS results from FinnGen and UK Biobank. After applying HEIDI filtering (p_HEIDI > 0.05) and FDR correction, we identified 189 probes with significant associations in the discovery group. Of these, 39 remained significant in the replication group (CCS, OR 1.3001, 95% CI 1.1226-1.5057, P < 0.01; see **Supplementary Table S4**). The SMR estimates in **Figures 2A, B** show similar effect directions and largely overlapping confidence intervals across FinnGen and UK Biobank. This supports the idea that these expression signals can cause CTS risk. These repeated associations together show a small group of blood-derived regulatory genes (HLA-DRB5, ARG1, IER3, CLOCK, ZBTB34, SLC39A13, MAFF, STX4) whose changed expression is likely to directly affect the likelihood of CTS.

To extend these transcriptome-level findings, we next evaluated circulating protein levels using pQTL-based Mendelian randomization across 4,907 plasma proteins from the deCODE study. Six proteins—CCS, HEXIM2, HEXIM1, CPZ, PTPN9, and SEMA3G—demonstrated significant associations with CTS in FinnGen and retained directionally concordant effects in UK Biobank (**Supplementary Table S5**). To ensure the robustness of the findings, the FDR approach was used to account for multiple comparisons (**Supplementary Table S5**). **Figures 2C, D** shows that higher levels of HEXIM2 and HEXIM1 in the blood are linked to a higher risk of CTS, whereas CPZ, PTPN9, and SEMA3G are linked to a lower risk. These patterns suggest that specific protein pathways can either cause or stop the development of CTS, depending on how they are regulated. Across both cohorts, heterogeneity remained low, and no horizontal pleiotropy was detected, supporting the conclusion that circulation-derived proteins—particularly CCS and the HEXIM family—play a causal and biologically coherent role in CTS development.

### 3.2 Colocalization analysis highlights CCS as a convergent gene-protein signal for CTS

**Figure 3:**
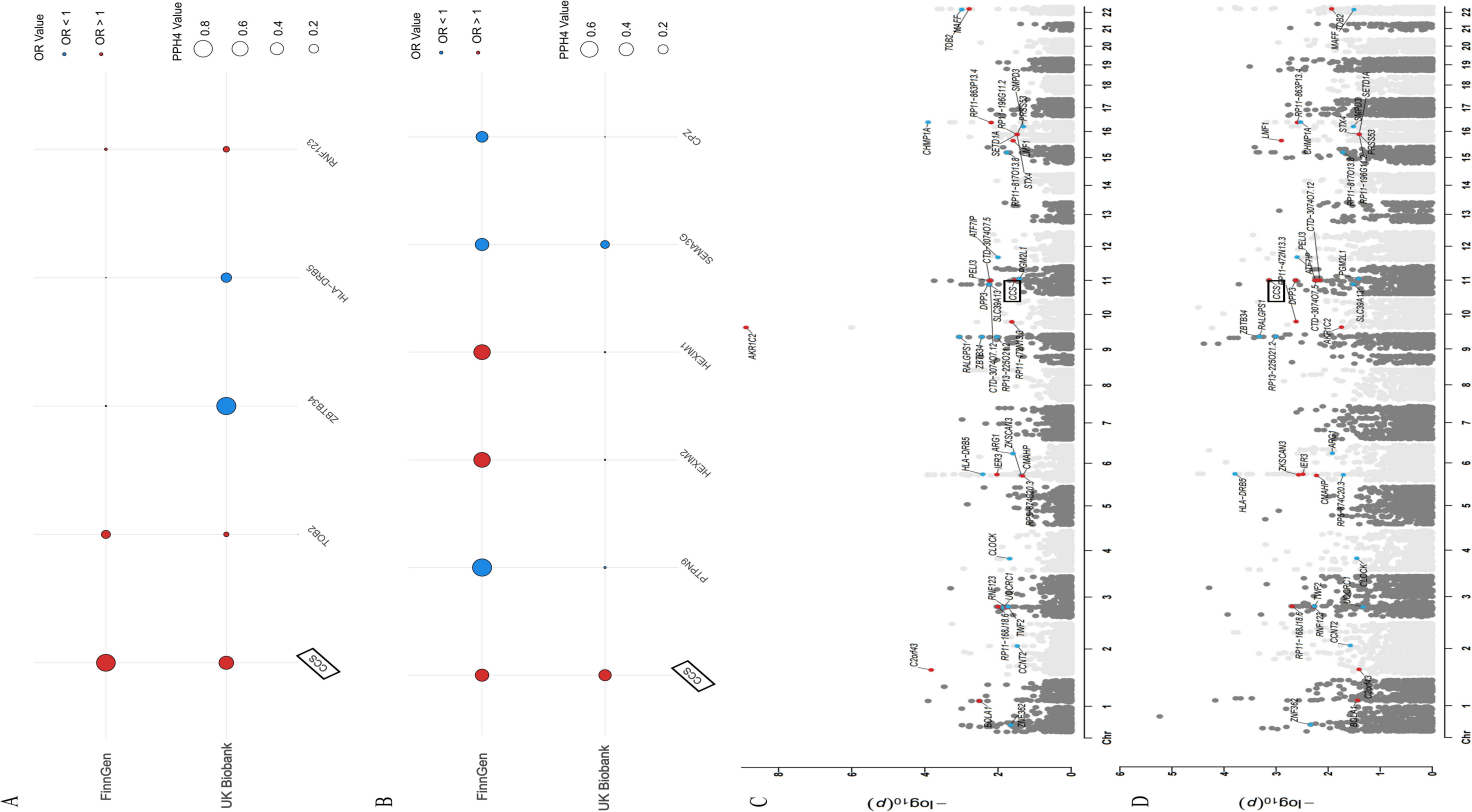
Colocalization support across cohorts and genome-wide association signals for CTS. **(A)** Colocalization probabilities for eQTL-CTS overlap in FinnGen and UK Biobank. Circle size reflects posterior support. CCS shows the strongest and most consistent colocalization across cohorts, whereas TOB2, ZBTB34, HLA-DRB5, and RNF123 display cohort-dependent signals. **(B)** Colocalization probabilities for pQTL-CTS overlap in FinnGen and UK Biobank. CCS again demonstrates the most robust support, whereas HEXIM1, HEXIM2, CPZ, and PTPN9 exhibit variable but concordant patterns. **(C)** Manhattan plot of CTS GWAS results in FinnGen, with key loci highlighted, including CCS, HLA-DRB5, TOB2, MAFF, and AKR1C2. **(D)** Manhattan plot of CTS GWAS results in UK Biobank, showing reproducible association peaks at corresponding loci.

We next applied Bayesian colocalization to determine whether the same causal variants jointly drive gene or protein levels and CTS risk. At the gene-expression level, COLOC supported that the 39 replicated SMR probes were associated with CTS in FinnGen (PPH3+PPH4 ≥ 0.80), and in these SMR probes, CCS shared causal variants with CTS (PPH4 = 0.8327), whereas shares moderate causal variants with CTS in UK Biobank (PPH4 = 0.50)(**Supplementary Table S6**). **Figure 3A** shows how the support for CCS is consistent across different groups, whereas the other genes only have support in specific groups or weaker support. The genome-wide Manhattan plots for CTS in FinnGen and UK Biobank (**Figures 3C, D**) show that these signals come from regions with clearly visible GWAS peaks.

At the protein level, COLOC analysis supported an association for 8 out of the 26 proteins in FinnGen (PPH3+PPH4 ≥ 0.80). Building on this, the CCS for these proteins was further demonstrated to be moderately associated with CTS in the UK Biobank (**Figures 3B**, **Supplementary Table S7**). Notably, CD14 and ANGPTL4 showed strong colocalization in FinnGen and weaker but consistent support in UK Biobank, whereas PTPN9 displayed only modest evidence despite significant MR associations. To ensure a comprehensive analysis, the threshold (PPH3+PPH4 ≥ 0.80) was utilised to expand the inclusion criteria. Of the selected genes, CCS shared the most significant causal variants with CTS (PPH4 = 0.8327). While colocalization evidence for CCS was relatively weak and variable across replicates (gene expression levels with CTS from the UK Biobank and proteomic datasets), it still appeared more prominent than that of other candidates. In contrast, eQTL analyses demonstrated relatively robust colocalization results for CCS probes.

Taken together, these colocalization results indicate that circulating levels and blood expression of CCS are likely driven by the same causal variants that underlie CTS risk. Integration of proteomic MR with COLOC therefore points to CCS-centred post-transcriptional mechanisms and highlights CCS as a particularly compelling protein-level biomarker and therapeutic target for CTS.

### 3.3 Transcriptomic and histopathological alterations in CTS

**Figure 4:**
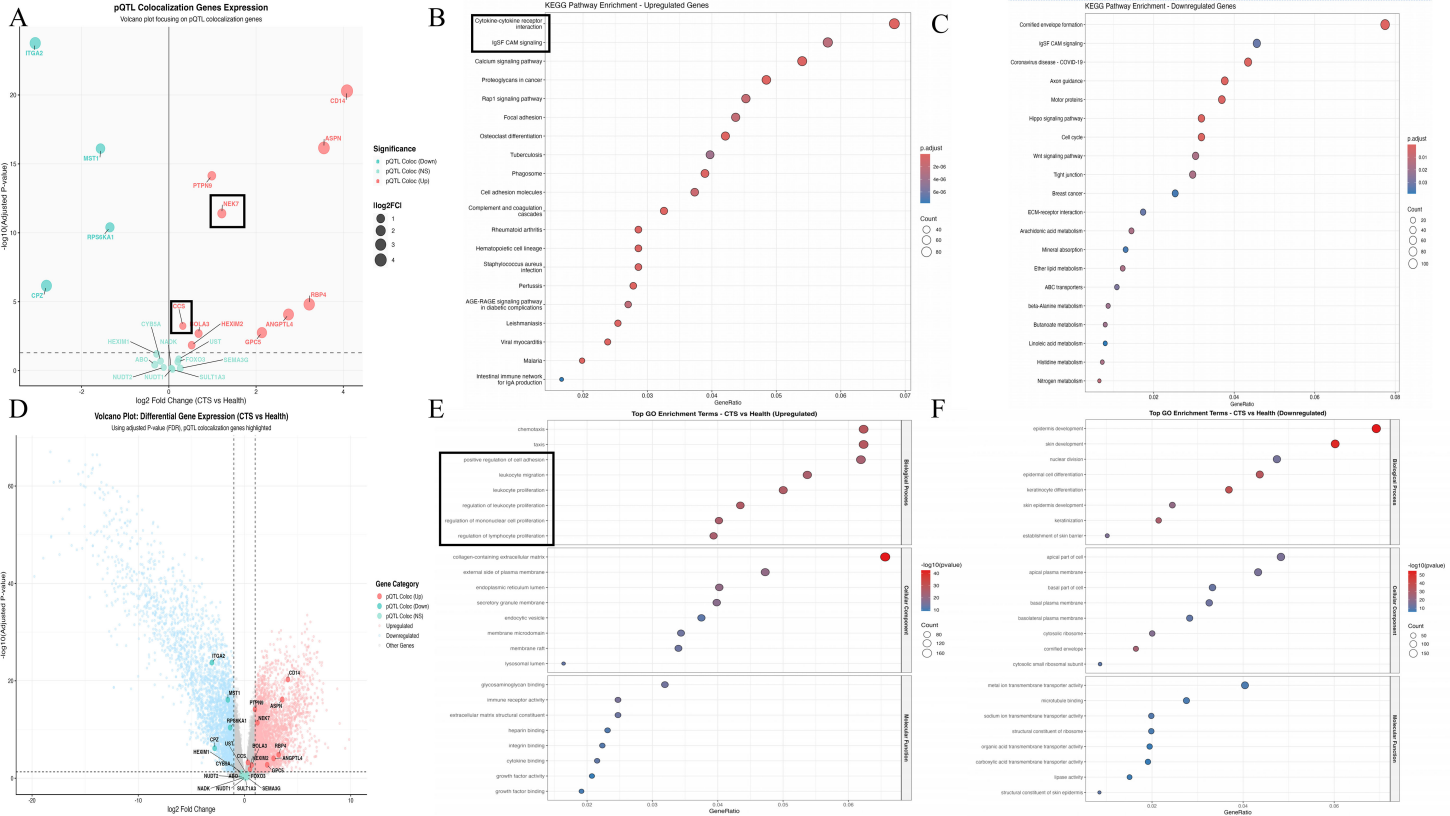
Transcriptomic alterations and pathway enrichment in CTS versus healthy controls. **(A)** Volcano plot showing differential expression of pQTL-colocalised genes in CTS versus healthy controls. Upregulated genes (red) include NEK7, ANGPTL4, and ASPN, whereas downregulated genes (blue) include MST1 and RPS6KA1. **(B)** KEGG enrichment of upregulated DEGs, highlighting cytokine–cytokine receptor interaction, IL-17 signalling, Rap1 signalling, osteoclast differentiation, and cell adhesion pathways. **(C)** KEGG enrichment of downregulated DEGs, showing reduced activity in Hippo signalling, ABC transporters, lipid and nitrogen metabolism, and ion-transport processes. **(D)** Global volcano plot of all DEGs, with pQTL-colocalised genes marked to illustrate overlap between genetic and transcriptional signals. **(E)** GO enrichment of upregulated DEGs, indicating enhanced leukocyte activation, cytokine-mediated signalling and collagen-containing extracellular matrix pathways. **(F)** GO enrichment of downregulated DEGs, demonstrating suppression of epidermal development, keratinocyte differentiation, structural molecule activity, and ion-transporter functions.

We next examined transcriptomic changes in carpal tunnel tissue to validate the genetic and proteomic findings at the organ level. Bulk RNA-seq comparing CTS samples with healthy controls identified a large number of DEGs using FDR < 0.05 and |log_2_FC| > 1. The volcano plots highlight prominent upregulation of NEK7, ANGPTL4, ASPN, and other pQTL-colocalised genes, together with downregulation of MST1, RPS6KA1, and several structural or metabolic genes (**Figures 4A, D**). Many of these dysregulated transcripts, including NEK7, ANGPTL4, and CCS, overlapped with SMR/COLOC-supported loci, indicating that genetically inferred causal genes also show consistent expression shifts in CTS tissue.

Pathway enrichment analysis of upregulated DEGs revealed a transcriptional program dominated by immune activation and extracellular matrix (ECM) remodelling. KEGG analysis showed significant enrichment of cytokine–cytokine receptor interaction, IL-17 signalling, Rap1 signalling, osteoclast differentiation, and cell adhesion molecule pathways (**Figure 4B**). GO biological process terms were enriched for leukocyte activation and proliferation, as well as for positive regulation of cell adhesion and cytokine-mediated signalling. In contrast, cellular component and molecular function categories emphasised collagen-containing ECM and cytokine/chemokine receptor binding (**Figure 4E**). In contrast, downregulated DEGs mapped to Hippo signalling, ABC transporters, lipid and nitrogen metabolism, and multiple ion-transport pathways (**Figure 4C**), with GO terms indicating suppressed epidermal development, keratinocyte differentiation, structural molecule activity, and ion transmembrane transporter function (**Figure 4F**).

### 3.4 Phenome-wide MR analysis identifies CCS as a CTS-specific causal protein and establishes GDM as an independent upstream driver

**Figure 5:**
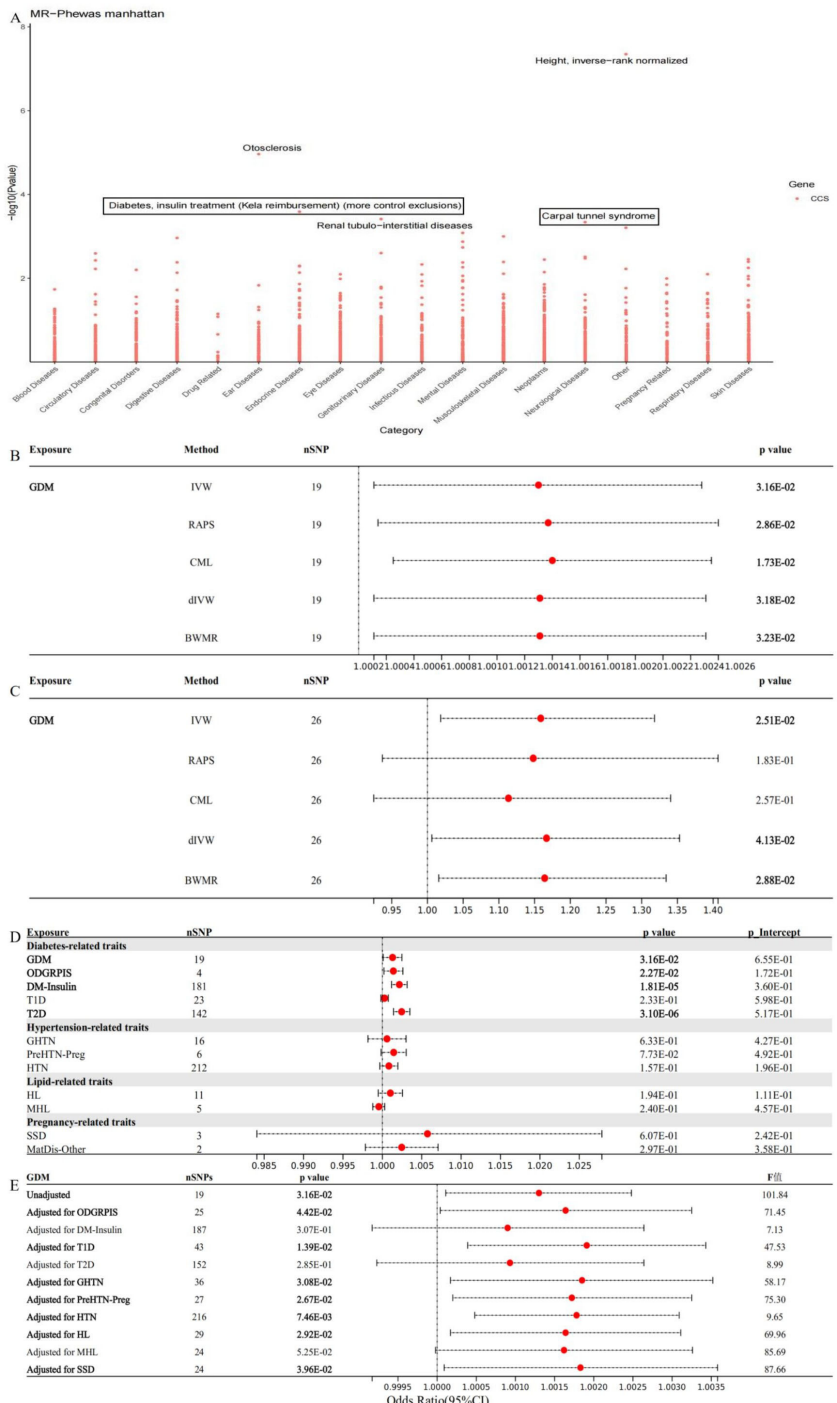
Phenome-wide and pathway-specific MR analyses linking GDM, CCS, and CTS. **(A)** MR-PheWAS Manhattan plot showing the association of circulating CCS levels with 2,454 FinnGen traits. Each point represents one outcome, with −log10(P) on the y-axis. CCS demonstrates its strongest associations with carpal tunnel syndrome, insulin-treated diabetes, renal tubulo-interstitial disorders, otosclerosis, and height. **(B)** Forest plot of the causal effect of gestational diabetes mellitus (GDM) on CTS in the discovery cohort using five MR methods (IVW, RAPS, CML, dIVW, and BWMR). All methods show directionally consistent odds ratios above 1 with significant P values. **(C)** Replication analysis in UK Biobank, again showing consistent positive MR estimates across methods, supporting the robustness of the GDM–CTS association. **(D)** Univariable MR estimates for diabetes-related, hypertension-related, lipid-related, and pregnancy-related traits on CTS. GDM and insulin-treated diabetes exhibit significant positive associations, whereas most other traits show weaker or non-significant effects. **(E)** Multivariable MR estimates for GDM on CTS after sequential adjustment for correlated metabolic and pregnancy traits. The direct GDM effect remains significant across models with adequate instrument strength, indicating that GDM contributes to CTS risk independently of related factors. IVW, inverse-variance weighted; RAPS, robust adjusted profile score; CML, constrained maximum likelihood; dIVW, debiased inverse-variance weighted; BWMR, Bayesian weighted MR; SNP, single-nucleotide polymorphism; OR, odds ratio; CI, confidence interval; GDM, gestational diabetes mellitus; CTS, carpal tunnel syndrome; T2D, type 2 diabetes; T1D, type 1 diabetes; ODGRPIS, other disorders of glucose regulation and pancreatic internal secretion; DM-Insulin, insulin-treated diabetes; HTN, hypertension; GHTN, gestational hypertension; PreHTN-Preg, pre-existing hypertension complicating pregnancy; HL, hyperlipidaemia; MHL, mixed hyperlipidaemia; SSD, single spontaneous delivery; MatDis-Other, other maternal disorders predominantly related to pregnancy.

To clarify the broader disease context of CCS, we identified the only candidate supported at both the transcriptomic (eQTL/COLOC) and proteomic (pQTL/MR/COLOC) levels. We performed a comprehensive MR-PheWAS across 2,454 phenotypes in FinnGen. The MR-PheWAS Manhattan plot demonstrated that CCS protein levels exhibit the potent and specific causal association with CTS (OR 1.3001; 95% CI 1.1226-1.5057; P < 0.01), with additional, though weaker, associations observed for diabetes requiring insulin treatment, renal tubulo-interstitial diseases, otosclerosis, and height (**Figures 5A**, **Supplementary Table S8**). The clustering of CCS effects within metabolic, renal, and peripheral neurological traits suggests that CCS biology intersects with systemic stress pathways, but its peak signal in CTS points toward a localised pathogenic role in the carpal tunnel microenvironment.

We next quantified the causal effect of gestational diabetes mellitus (GDM), a clinical state strongly associated with oxidative stress and microvascular dysfunction, on CTS risk. In the discovery cohort, all five MR methods (IVW, RAPS, CML, dIVW, and BWMR) demonstrate that GDM increases CTS susceptibility. In the replication cohort, three MR methods (IVW, dIVW, and BWMR) also indicated that GDM is a risk factor for CTS. Consistent with this, other methods (RAPS and CML) produced directionally concordant odds ratios, although these did not reach statistical significance.

We then surveyed a broad range of diabetes-related, hypertension-related, lipid-related, and pregnancy-related traits using univariable MR. Only GDM and diabetes requiring insulin therapy showed robust positive associations with CTS. Finally, we performed multivariable MR to evaluate whether the GDM–CTS relationship persists after adjusting for correlated traits. Across all adjustment models—including those correcting for other glycaemic disorders, T1D/T2D, hypertension phenotypes, and lipid levels—the direct effect of GDM on CTS remained significant with adequate instrument strength, indicating that GDM independently contributes to CTS risk rather than acting through shared metabolic pathways.

Together, these findings demonstrate that CCS is a disease-specific causal protein for CTS and that GDM is a key upstream determinant that increases CTS risk independently of other metabolic or obstetric traits. The convergence of MR-PheWAS and GDM-focused MR analyses (The specific analytical procedure is presented in Results section 5.) supports a mechanistic model in which hyperglycaemic pregnancy elevates circulating CCS levels, thereby promoting a pro-oxidative, ECM-remodelling microenvironment in the carpal tunnel that favours nerve compression.

### 3.5 Gestational diabetes mellitus exerts an independent, CCS-mediated causal effect on CTS

We first evaluated whether GDM and CTS share a common genetic background. Linkage disequilibrium score regression using FinnGen and UK Biobank summary statistics showed a significant positive genetic correlation between the two traits (rg = 0.219, SE = 0.061, P = 3.52×10^-4^), indicating that overlapping common variants contribute to both conditions and providing a rationale for formal causal inference.

We then quantified the causal effect of GDM on CTS. In the primary MR analysis, we used FinnGen GDM (18,581 cases/263,483 controls) as exposure and UK Biobank CTS (8,289 cases/454,721 controls) as outcome, selecting 19 independent SNP instruments (p < 5×10^-8^, LD r² < 0.001) after BMI adjustment. Across IVW, RAPS, CML, dIVW, and BWMR, all methods yielded directionally consistent odds ratios above 1 with statistically significant P values (**Figure 5B**; **Supplementary Tables S9-S11**). Additionally, the Steiger test result between GDM and CTS was TRUE, suggesting without an inverse causal link (p < 0.05). The IVW estimate indicated that genetically increased liability to GDM raises CTS risk (OR = 1.0013; 95% CI 1.0001-1.0025; P < 0.05), with low heterogeneity (Q = 17.02, P = 0.52) and no evidence of horizontal pleiotropy (Egger intercept = 0.0000582, P = 0.66).

Because GDM is strongly sex-biased, we repeated the MR analysis in women only. Using female-restricted UK Biobank data, 26 GDM-associated SNPs remained strong instruments (F statistic 22.7; **Supplementary Table S14**). CTS data were from FinnGen as the outcome (31,181 CTS cases/435,371 controls). The sex-stratified IVW analysis showed a 15.9% higher CTS risk per unit increase in GDM genetic liability in women (OR 1.1586; 95% CI 1.0185-1.3179; P = 0.03; **Figure 5C**; **Supplementary Tables S12-S13**), with no detectable heterogeneity or directional pleiotropy (Q = 30.74, P = 0.2; Egger intercept = −0.01, P = 0.41). Furthermore, the Steiger directionality test supported the proposed causal direction (Steiger test: TRUE; p < 0.05). This effect size exceeded that observed in sex-combined analyses (OR = 1.0013; 95% CI 1.0001-1.0025; P < 0.05), suggesting that pregnancy-related physiological changes may amplify the impact of GDM on median nerve compression. In the cohort, dIVW and BWMR also indicated that GDM is a risk factor for CTS. Moreover, other methods (RAPS and CML) produced directionally concordant odds ratios, though with no statistical significance.

To place GDM in the context of related cardiometabolic traits, we performed univariable MR for a panel of diabetes-, hypertension-, lipid-, and pregnancy-related exposures. Diabetes traits such as “other disorders of glucose regulation and pancreatic internal secretion” (ODGRPIS), insulin-treated diabetes (DM-Insulin), and type 2 diabetes (T2D) all showed significant positive associations with CTS, whereas type 1 diabetes (T1D) did not (**Figure 5D**; **Supplementary Table S15**). Traits related to blood pressure, lipids, and pregnancy complications (including gestational hypertension, pre-existing hypertension in pregnancy, hyperlipidaemia, and single spontaneous delivery) had weak or non-significant effects. Multivariable MR models that simultaneously adjusted GDM for ODGRPIS, T1D, GHTN, PreHTN-Preg, HTN, HL, and SSD showed that the direct GDM–CTS effect remained positive and statistically significant in models with adequately strong instruments (F > 10) (**Figure 5E**; **Supplementary Table S16**). Where attenuation occurred, instrument strength was suboptimal, suggesting possible weak instrument bias rather than the absence of effect. Overall, these results indicate that GDM confers CTS risk independently of other metabolic, hypertensive, and pregnancy-related traits.

Finally, we examined whether CCS mediates the causal pathway from GDM to CTS using a two-step MR mediation framework. In the first step, genetic liability to GDM significantly increased circulating CCS levels (β = a, P < 0.05), consistent with pQTL-based MR findings. In the second step, higher CCS levels were strongly associated with increased CTS risk (β = b, P < 0.001). The product of coefficients (a×b) yielded a significant indirect effect (β = 0.1004; 95% CI 0.0292-0.1716; P = 0.0051), corresponding to an OR of 1.106 (95% CI 1.030-1.187; P = 0.0051). The total effect of GDM on CTS was β = 0.1518 (OR 1.1640; 95% CI 1.0280-1.3180, P = 0.0166), whereas the direct effect after conditioning on CCS was substantially attenuated and no longer significant (β = 0.0514, 95% CI: −0.0918-0.1946; OR 1.0528, 95% CI: 0.9123-1.2149; **Supplementary Table S17**). CCS mediated an estimated 66.1% of the total GDM–CTS effect (95% CI 19.2%-113.0%), and the Sobel test confirmed a robust mediation signal (P = 0.0051).

**Figure 6:**
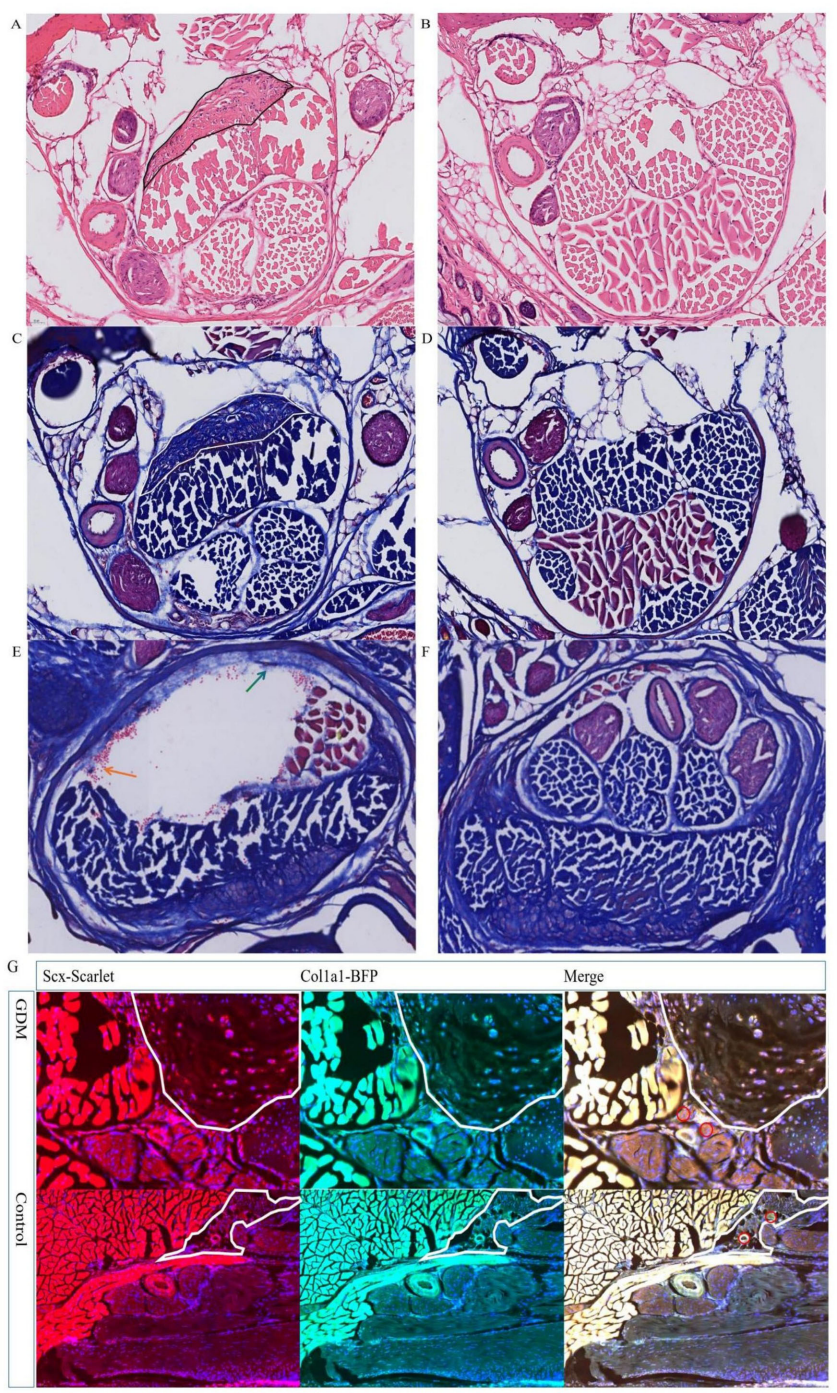
Histopathological changes in the carpal tunnel of CTS and control mice. **(A, B)** H&E staining of transverse carpal tunnel sections from CTS mice **(A)** and normoglycaemic pregnant controls **(B)**. CTS mice show a dense peri-tunnel inflammatory infiltrate and pronounced extracellular matrix (ECM) thickening along the tunnel wall. **(C, D)** Masson’s trichrome staining (10×) of CTS **(C)** and control **(D)** tunnels, demonstrating expansion of the peritendinous connective tissue in CTS mice. **(E, F)** Higher-magnification Masson images (20×) of CTS **(E)** and control **(F)** tissue. CTS mice exhibit prominent inflammatory cell infiltration around the tunnel (orange arrow) and collagen-rich ECM overgrowth (green arrow). **(G)** Immunofluorescence staining of wrist canal-associated connective tissue in GDM and control mice. GDM mice show marked peritendinous thickening with strong Scx and Col1a1 expression, whereas controls display minimal Col1a1 signal.

Taken together, these analyses show that GDM and CTS share a significant genetic correlation, that GDM exerts a reproducible and female-specific causal effect on CTS independent of other metabolic traits, and that a substantial proportion of this effect operates through elevations in circulating CCS. This supports a mechanistic model in which hyperglycaemic pregnancy upregulates CCS, thereby promoting oxidative stress and extracellular matrix remodelling in the carpal tunnel and predisposing to symptomatic nerve compression. Histopathological examination of the carpal tunnel provided concordant morphological evidence for these transcriptomic changes. H&E staining in CTS mice showed dense peri-tunnel inflammatory cell infiltration and pronounced thickening of the tunnel wall ECM, whereas normoglycaemic pregnant controls displayed a compact, well-organised tunnel structure (**Figures 6A, B**). Masson’s trichrome staining confirmed expansion of peritendinous connective tissue in CTS animals (**Figures 6C, D**), and higher-magnification images demonstrated that the infiltrating cells were blood-derived and that the expanded ECM consisted predominantly of collagen fibres (**Figures 6E, F**). Immunofluorescence staining showed that GDM mice had significant peritendinous thickening, with strong expression of Scx and Col1a1 (**Figure 6G**). Together, the RNA-seq and histological data indicate that CTS is characterised by an inflamed, collagen-rich, and metabolically impaired tissue microenvironment, within which genes supported by genetic data, such as NEK7, ANGPTL4, CCS, and related targets, are positioned at the intersection of immune activation and ECM remodelling.

### 3.6 Single-cell transcriptomics reveal CCS-high interferon-responding cells and altered trajectories under GDM

**Figure 7:**
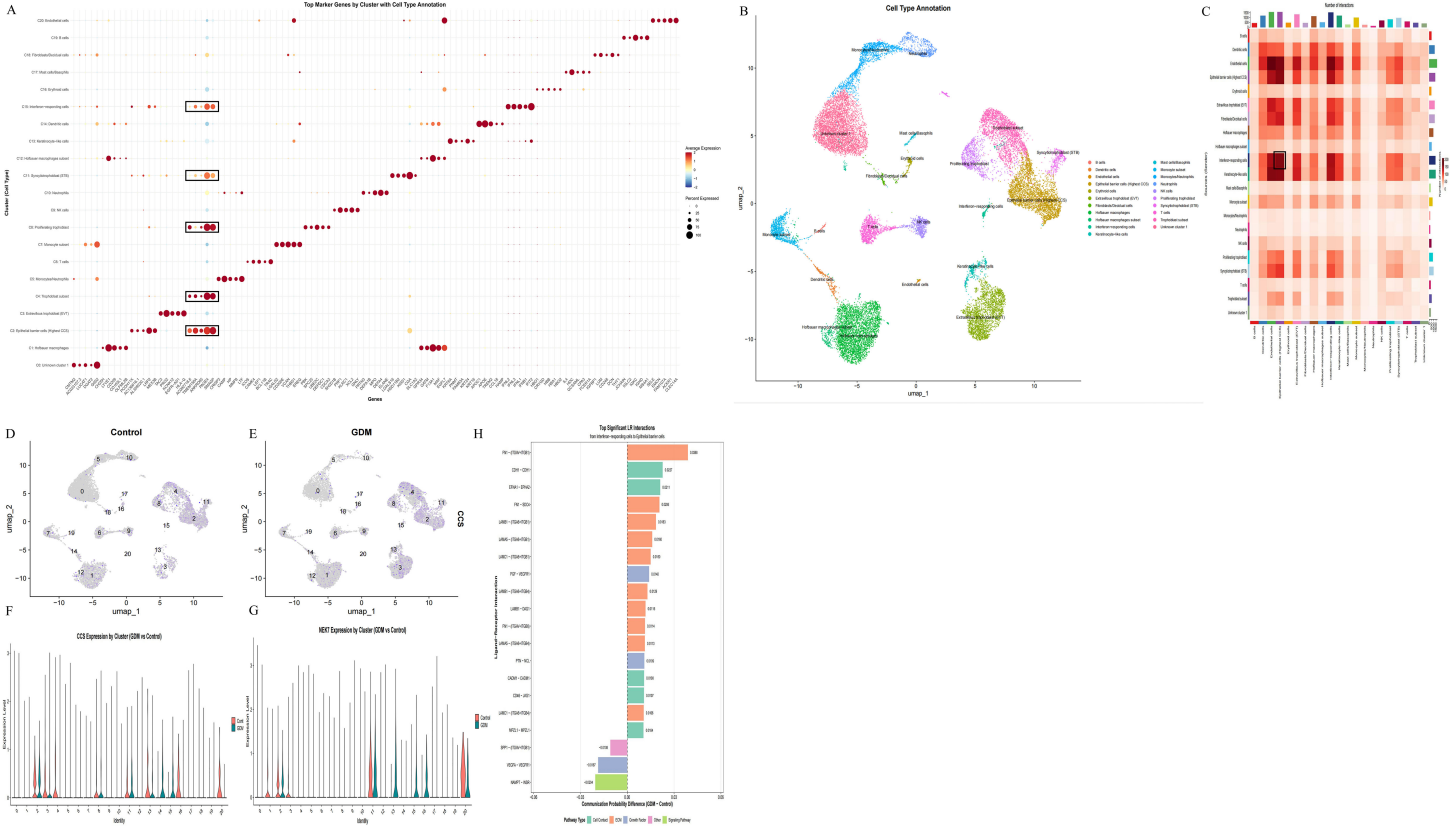
Single-cell transcriptomic landscape of placental cell from women with GDM and controls. **(A)** Dot plot showing top marker genes for each cluster, used to define major immune and stromal populations in the placental cell single-cell dataset. **(B)** UMAP visualization of annotated cell types, including B cells, T cells, NK cells, monocyte subsets, neutrophils, dendritic cells, Hofbauer macrophages, interferon-responding cells, and epithelial barrier cells. **(C)** Heatmap of inferred cell–cell communication, illustrating ligand–receptor interactions across clusters. Interferon-responding cells exhibit the largest increase in interactions with epithelial barrier cells, which show the highest CCS expression. **(D, E)** UMAP feature plots of CCS expression in control **(D)** and GDM **(E)** placental cell, demonstrating marked upregulation of CCS in interferon-responding cells under GDM. **(F)** Violin plots of CCS expression across clusters in control and GDM samples, confirming selective CCS induction in interferon-responding cells. **(G)** Violin plots of NEK7 expression across clusters, showing a similar GDM-associated increase in interferon-responding and myeloid populations. **(H)** Bar plot of the most significant ligand–receptor interactions from interferon-responding cells to epithelial barrier cells, indicating that GDM-induced enhancement of intercellular communication is concentrated in extracellular matrix-related pathways.

Using a public single-cell RNA-seq dataset of placental cell from women with GDM and normoglycaemic pregnant controls, we mapped the immune landscape of CTS-related genes at single-cell resolution. After standard quality control and UMAP embedding, we defined significant immune and stromal populations by cluster-specific marker genes, including B cells, T cells, NK cells, neutrophils, dendritic cells, monocytes and myeloid subsets, Hofbauer macrophages, interferon-responding cells, and epithelial barrier cells (**Figures 7A, B**). We then examined CCS expression across clusters. Feature and violin plots showed that CCS expression was low in most lineages but was selectively and markedly increased in interferon-responding cells from GDM samples, with only minimal signal in controls (**Figures 7D–F**). NEK7 displayed a similar pattern, with higher expression in interferon-responding cells and myeloid populations under GDM (**Figure 7G**). Cell–cell communication analysis demonstrated that interferon-responding cells established the strongest ligand–receptor interactions with epithelial barrier cells, which themselves expressed the highest CCS levels; these enhanced interactions were concentrated in extracellular-matrix-related pathways (**Figures 7C, H**).

**Figure 8:**
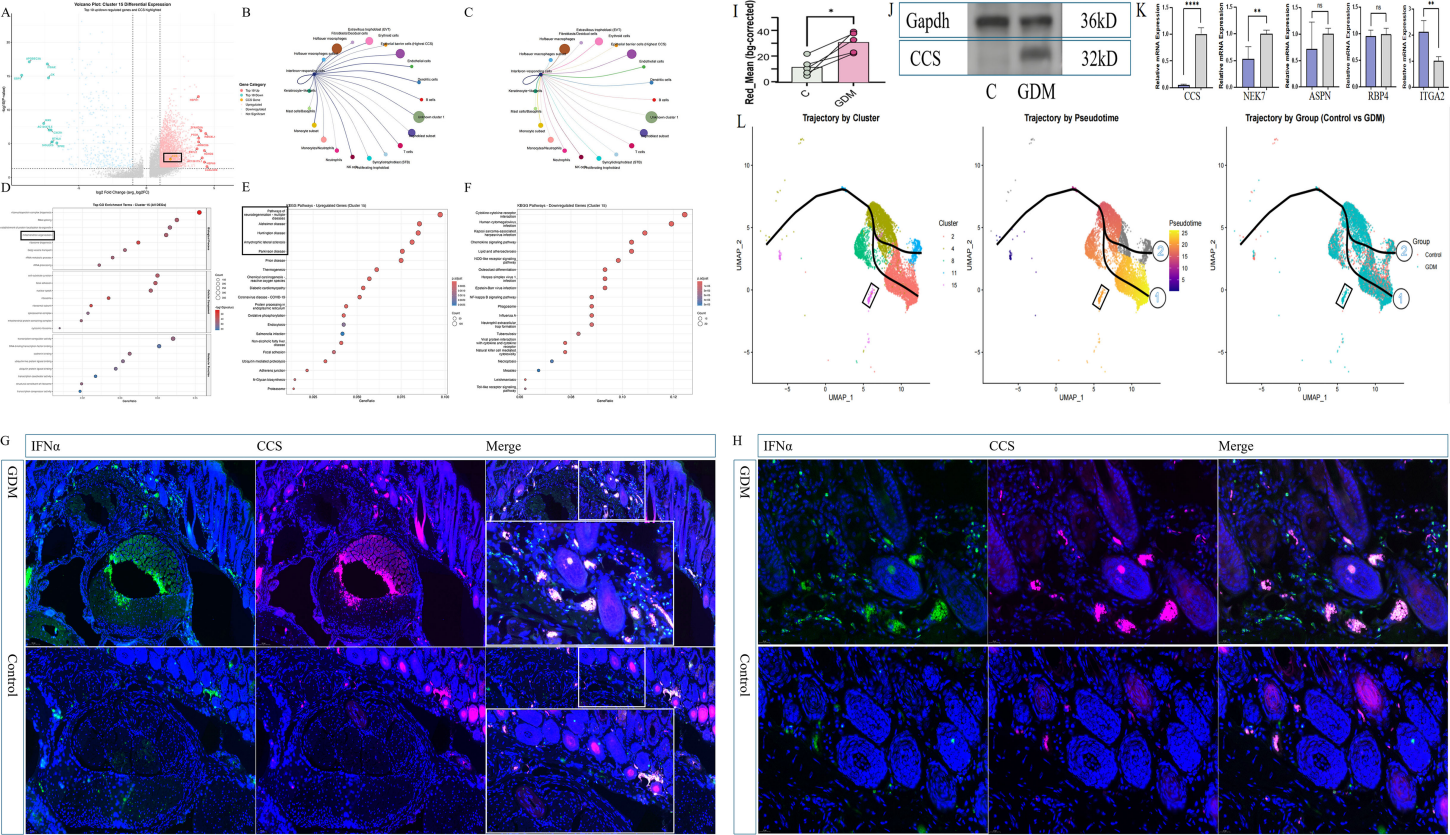
Functional enrichment of cluster 15 (interferon-responding cells), experimental validation of CCS upregulation in GDM, and trajectory inference. **(A)** Volcano plot showing differential expression within cluster 15, demonstrating a marked GDM-associated increase in CCS in interferon-responding cells. **(B, C)** Cell–cell communication analysis confirming that interferon-responding cells exhibit the strongest ligand–receptor interactions with epithelial barrier cells, consistent with patterns observed in **Figure 7C**. **(D)** GO enrichment of DEGs in cluster 15 (GDM *vs*. control), highlighting pathways related to immune activation, leukocyte proliferation, and cytokine-mediated signalling. **(E, F)** KEGG enrichment of upregulated and downregulated DEGs, respectively. Upregulated genes map to neurodegeneration-related pathways, oxidative phosphorylation, and chemokine signalling, whereas downregulated genes involve cytokine–cytokine receptor interaction, viral response pathways and NOD-like receptor signalling. **(G)** Immunofluorescence staining of wrist canal-associated connective tissue in GDM and control mice, showing pronounced peritendinous thickening and strong IFNα expression in GDM mice with minimal signal in controls. **(H)** Additional immunofluorescence images demonstrating replicable IFNα upregulation in GDM mouse tissue. **(I)** Background-corrected mean red fluorescence intensity of CCS staining (Red Mean, bg-corrected) in control **(C)** and GDM groups. Bars show group means (± SEM or ± SD), points denote individual experimental units, and connecting lines indicate paired measurements where applicable. *P < 0.05. **(J, K)** Western blot and qPCR analyses confirming elevated CCS expression in wrist-canal tendon tissue from GDM mice. NEK7 expression was also increased, whereas ASPN and RBP4 showed no group difference; ITGA2 showed modest upregulation. **(L)** Pseudotime trajectory analysis showing that GDM alters the developmental progression of interferon-responding cells. Control samples primarily followed pathway 2, whereas GDM shifted cells toward pathway 1, promoting differentiation toward epithelial barrier cell states. This trajectory shift aligns with increased epithelial-barrier interactions and CCS-associated signalling under GDM.

To further dissect the molecular state of interferon-responding cells, we focused on cluster 15. Differential expression analysis confirmed a robust GDM-associated increase of CCS in this cluster (**Figure 8A**). Cell–cell communication analyses again highlighted reinforced crosstalk between interferon-responding cells and epithelial barrier cells (**Figures 8B, C**). GO enrichment of cluster-15 DEGs revealed activation of immune-related processes, including leukocyte proliferation and cytokine-mediated signalling. At the same time, KEGG analysis showed upregulation of neurodegeneration-related pathways, oxidative phosphorylation, and chemokine signalling, with concomitant downregulation of cytokine–cytokine receptor interaction, viral infection pathways, and NOD-like receptor signalling (**Figures 8D–F**). Pseudotime trajectory analysis indicated that GDM reshaped cell-fate dynamics: In controls, interferon-responding cells predominantly followed trajectory path 2, whereas in GDM, they were redirected along path one towards an epithelial barrier cell-like state (**Figure 8L**). This trajectory shift, together with strengthened ECM-focused communication, supports a model in which GDM drives expansion and reprogramming of CCS-high interferon-responding niches that fuel epithelial barrier cell activation.

We validated these single-cell observations in a GDM mouse model. Immunofluorescence staining of wrist-canal-associated connective tissue showed pronounced peritendinous thickening and intense CCS signal in GDM mice, in contrast to the thin connective tissue and weak CCS staining in normoglycaemic pregnant controls (**Figure 8G**). Tests on the tissue from the wrist showed that there was a clear increase in the levels of CCS protein and mRNA in mice with GDM (**Figures 8J, K**). NEK7 expression increased in parallel, whereas ASPN and RBP4 did not differ between groups, and ITGA2 displayed only a slight increase (see **Figures 8H, I**). Together, these data indicate that GDM systemically and locally induces a CCS-high interferon-responding cell program, enhances ECM-related communication with epithelial barrier cells and increases CCS expression in carpal-tunnel-associated tissues, providing a mechanistic cellular link between GDM, CCS dysregulation, and CTS susceptibility.

### 3.7 Druggability of CTS-related candidates supported by molecular docking

**Figure 9:**
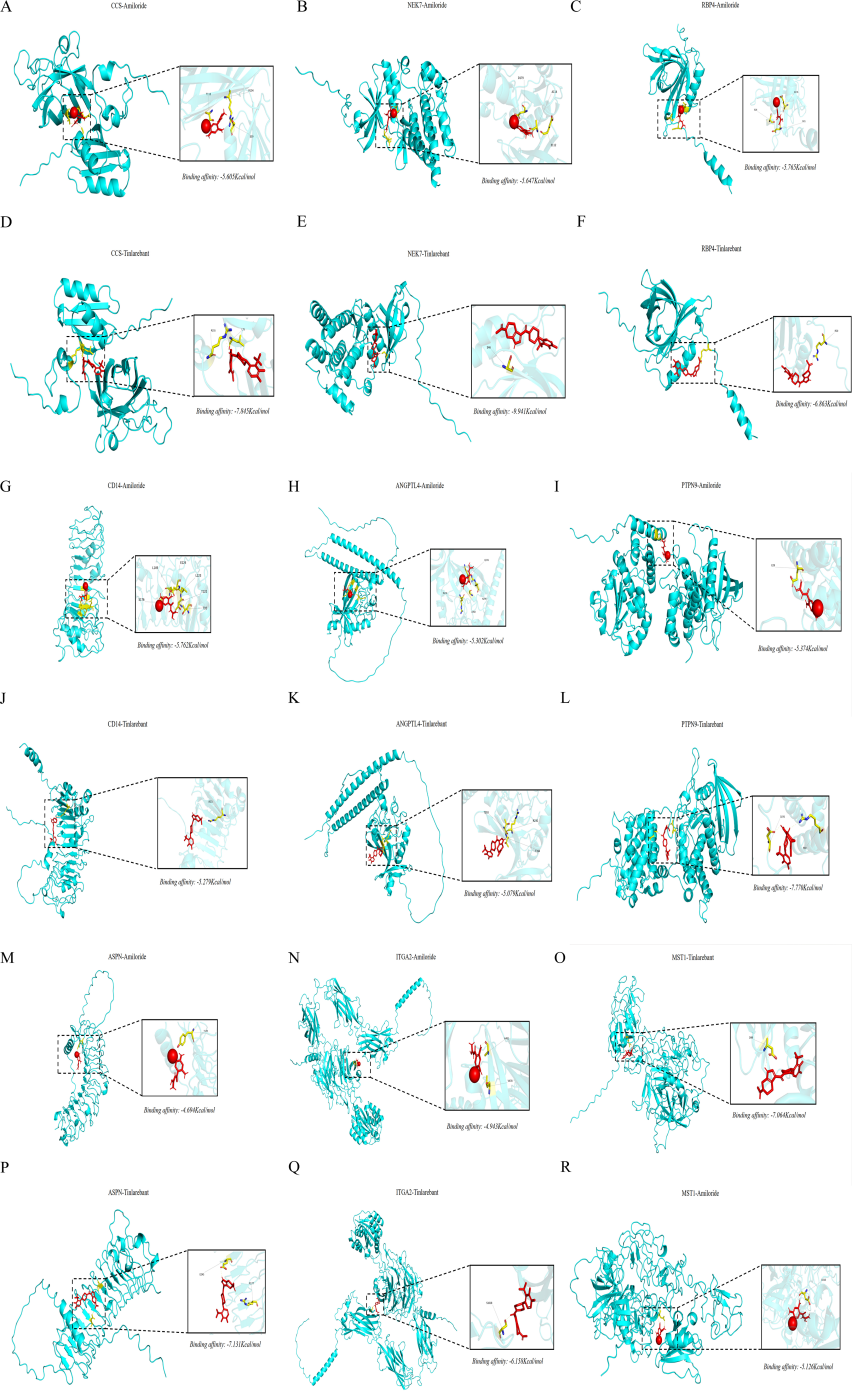
Molecular docking analysis of candidate CTS-related proteins with Amiloride and Tinlarebant. **(A–C)** Docking conformations of CCS, NEK7, and RBP4 with Amiloride, each showing stable ligand–protein interactions (binding energies −5.60, −5.47, and −5.76 kcal/mol, respectively). **(D–F)** Docking of the same proteins with Tinlarebant demonstrated stronger interaction energies, particularly for CCS (−7.85 kcal/mol) and NEK7 (−9.94 kcal/mol), indicating enhanced binding stability relative to Amiloride. **(G–I)** Binding modes of Amiloride with CD14, ANGPTL4, and PTPN9, all showing meaningful affinities (−5.30 to −5.76 kcal/mol). **(J–L)** Tinlarebant showed similarly favourable or stronger interactions with these targets, including high-affinity binding to PTPN9 (−7.77 kcal/mol). **(M–O)** Docking with ECM-associated proteins ASPN, ITGA2, and MST1 showed variable affinities, with MST1–Tinlarebant (−7.06 kcal/mol) representing one of the strongest interactions across all protein–ligand pairs. **(P–R)** Additional docking views confirm stable ligand positioning within protein binding pockets, supporting the robustness of the docking predictions.

Through screening the DrugBank, Therapeutic Target Database, ChEMBL, DGIdb, and PharmSnap databases for inhibitors/activators with CTS, the inhibitors, Amiloride (Aco1) was identified, whereas, screening the databases for 26 proteins (pQTL), Tinlarebant (RBP4) was identified. To evaluate the therapeutic tractability of CTS-related proteins identified by our multi-omics analyses, we performed structure-based molecular docking of two clinically available small molecules, Amiloride and Tinlarebant, against a panel of 26 proteins (pQTL); we found that CCS, NEK7, RBP4, CD14, ANGPTL4, PTPN9, ASPN, ITGA2, and MST1 were indicative of a favourable interaction. After standard protein structure preprocessing and energy minimization, we considered binding free energies below −5.0 kcal/mol as indicative of a favourable interaction. Both compounds formed stable complexes with multiple targets (**Figures 9A–R**). Amiloride showed meaningful affinity for CCS (−5.60 kcal/mol), NEK7 (−5.47 kcal/mol), and RBP4 (−5.76 kcal/mol) and similarly engaged CD14, ANGPTL4, and PTPN9 with binding energies between −5.30 and −5.76 kcal/mol. Tinlarebant generally produced stronger docking scores, with markedly enhanced affinities for CCS (−7.85 kcal/mol) and NEK7 (−9.94 kcal/mol), as well as robust binding to PTPN9 (−7.77 kcal/mol). Docking to ECM-associated proteins revealed variable but often favourable interactions; in particular, MST1-Tinlarebant (−7.06 kcal/mol) ranked among the strongest protein–ligand pairs. In the docking models, both ligands were found to be located in specific binding pockets and formed multiple hydrogen bonds and hydrophobic interactions with important functional residues. These results show that the docking experiments are reliable and that amiloride, and especially Tinlarebant, can form stable complexes with several CTS-related targets.

These *in silico* findings align with and extend our genetic and transcriptomic data to define a CCS-centred, druggable network. CCS, the only candidate supported simultaneously by eQTL, pQTL, colocalization, and mediation MR analyses, emerges as a desirable target: Strong binding to both Amiloride and Tinlarebant suggests that its copper-binding region is amenable to small-molecule modulation, potentially enabling restoration of copper homeostasis and attenuation of oxidative stress in the median nerve microenvironment. NEK7 and PTPN9 also exhibit high-affinity binding to Tinlarebant, suggesting that pharmacological interference with inflammasome activation and phosphatase-mediated signalling could provide additional benefit. RBP4, CD14, and ANGPTL4 link druggability to lipid transport and innate immune pathways, whereas ASPN, ITGA2, and MST1 connect small-molecule binding to extracellular matrix remodelling and Hippo signalling. Collectively, these results indicate that several components of the GDM–CCS–CTS axis are chemically addressable and nominate Amiloride and, in particular, Tinlarebant as promising scaffolds for mechanism-based prevention or treatment strategies targeting CTS in women with gestational diabetes.

## 4 Discussion

We integrated bidirectional, multivariable, and mediation Mendelian randomization (MR) with multi-omics (eQTL and pQTL) analyses to test whether gestational diabetes mellitus (GDM) causally increases the risk of carpal tunnel syndrome (CTS). Bidirectional MR showed that genetic liability to GDM consistently raised CTS risk, whereas reverse MR did not support CTS as a cause of GDM. Multivariable MR further confirmed that this effect was independent of other diabetes-related, hypertensive, lipid-related, and pregnancy-specific biomechanical traits. In two-step mediation MR, circulating CCS protein levels explained approximately two-thirds of the total causal effect of GDM on CTS, indicating that a significant component of GDM-related risk operates through CCS-driven pathways of oxidative injury and metal–ion imbalance (57). These findings support the need for strict glycaemic control and targeted neurological assessment in women with GDM, particularly in late pregnancy when fluid retention and metabolic derangements may act together to increase median nerve vulnerability.

At the molecular level, chronic inflammation, oxidative stress, and fibrosis are recognised contributors to CTS pathophysiology (58). Our integrative SMR and Bayesian colocalization analyses in whole blood identified five genes—TOB2, ZBTB34, HLA-DRB5, RNF123, and CCS—with strong, replicated causal and colocalised signals across FinnGen and UK Biobank. HLA-DRB5, a key component of the MHC-II antigen-presentation pathway, links CTS risk to immune-mediated inflammation and adaptive immune activation (59). ZBTB34 also showed robust replication and colocalization, consistent with a role in transcriptional control of immune and fibrotic responses. These transcriptomic signals, together with bulk RNA-seq and histopathology showing immune-cell infiltration and collagen-rich matrix expansion in CTS tissue, implicate immune dysregulation, antigen presentation, and chronic inflammation as central mechanisms linking metabolic disturbance in GDM with structural remodelling in the carpal tunnel.

Proteomic analyses reinforced and refined this mechanistic framework. pQTL-based MR and colocalization identified several circulating proteins—including CCS, HEXIM2, HEXIM1, CPZ, and PTPN9—as causally associated with CTS. CCS occupied a unique position: It was supported by convergent evidence at both the eQTL and pQTL levels, and phenome-wide MR (MR-PheWAS) demonstrated that its strongest and most specific causal association was with CTS rather than with a broad spectrum of metabolic or inflammatory traits. CCS controls how copper is handled within cells and maintains redox balance (57). In this case, our data support a disease model in which problems with copper levels and associated oxidative stress within the carpal tunnel contribute significantly to CTS pathogenesis, especially in cases of hyperglycaemia in pregnancy. The single-cell data further showed that GDM expands CCS-high interferon-responding cell niches and redirects their trajectories toward epithelial barrier cells with intensified extracellular matrix signalling (60), offering a cellular explanation for how CCS links systemic metabolic stress to local nerve compression.

The druggability evaluation of genes emerging from SMR and COLOC analyses suggests several translational opportunities. HLA-DRB5 is already embedded within well-characterised immunomodulatory pathways, suggesting that targeting MHC-II-related mechanisms could temper local inflammation in CTS (59). In contrast, TOB2, ZBTB34, and RNF123 currently lack approved modulators but represent attractive candidates for future drug discovery targeting immune and fibrosis pathways (61). At the protein level, molecular docking indicated that clinically available small molecules, such as Amiloride and, especially, Tinlarebant, can stably bind CCS and other CTS-related proteins, including NEK7 and PTPN9, suggesting that pharmacological modulation of copper homeostasis, inflammasome activation, and phosphatase signalling is technically feasible. These observations open avenues for precision prevention and mechanism-based therapies in metabolically vulnerable women, including those with GDM.

This work has several strengths. We used complementary MR frameworks (bidirectional, multivariable, mediation) and integrated large-scale eQTL and pQTL resources from two independent European cohorts, applying stringent Bayesian colocalization criteria (PPH4 ≥ 0.80) to prioritise credible causal genes and proteins. However, at the proteomic level, evidence for CCS colocalization was weaker and less consistent across datasets. The pQTL results showed greater variability, with findings from the Discovery cohort (FinnGen) not being replicated in the Replication cohort. This discrepancy is likely due to population bias. Moreover, we only looked at people whose ancestors came from Europe. This means that the results might not be relevant to other ethnic groups (62). The disparity in results between the discovery and replication cohorts may be attributed to the limited number of genome-wide significant IVs for GDM. In the discovery cohort, all five MR methods (IVW, RAPS, CML, dIVW, and BWMR) consistently identified GDM as a risk factor for CTS. However, in the replication cohort, only three methods (IVW, dIVW, and BWMR) yielded statistically significant results, whereas the other two methods (RAPS and CML) produced directionally concordant effect estimates that were not statistically significant. This discrepancy was likely due to the susceptibility of different MR methods to weak-instrument bias when the number of strong genetic instruments is modest. Although measured effects are sourced from various tissues (blood eQTLs, circulating pQTLs, placenta, connective tissues, etc.), they indicate that CCS is a significant target for genetic intervention in the CTS in pregnant women with GDM. Critically, analysis of placental tissues supports a model that GDM drives the expansion and reprogramming of CCS-high, interferon-responsive niches. This process fuels the activation of epithelial barrier cells, enhances ECM-related communication, and elevates CCS expression in carpal-tunnel-associated tissues, thereby providing a mechanistic cellular link between GDM, CCS dysregulation, and CTS susceptibility. Although the study revealed a genetic-level association, residual confounding from pregnancy-related hormonal, biomechanical, or environmental factors cannot be entirely excluded. Furthermore, the scRNA-seq analysis used placental tissue. While the placenta is of foetal origin, it serves as the central interface of maternal–foetal communication. Thus, it is both exposed to and dynamically shaped by the maternal GDM environment. Furthermore, it is important to note a key limitation of our docking analysis. While it provides evidence that the candidate small molecules are able to bind to CCS, this does not confirm any modulatory functional effect on the protein’s activity. Future studies employing functional assays *in vitro* or in cellular models are essential to validate these predictions and assess therapeutic potential.

Moreover, this research should be conducted with other populations and include more information on the severity of GDM, its treatment, and blood sugar control. This should be combined with genetic and other types of data, as well as studies examining how the median nerve changes over time. This will help us better understand the causes of GDM.

In summary, our multi-layered MR and multi-omics analyses provide strong causal and mechanistic evidence that GDM increases CTS risk via CCS-centred metabolic and oxidative stress pathways. Additional immune and inflammatory signals, particularly those involving HLA-DRB5 and ZBTB34, broaden the mechanistic landscape and highlight new therapeutic targets. Clinically, these data support tighter glycaemic control, proactive CTS screening, and early intervention in pregnant women with GDM, and they lay the groundwork for precision strategies aimed at modulating CCS and related pathways to mitigate CTS in high-risk metabolic settings.

## 5 Conclusion

We combined bidirectional, multivariable, and mediation MR with multi-omics colocalization to demonstrate that GDM causally increases CTS risk. Two-step mediation MR pinpointed circulating CCS as a key intermediary, with approximately two-thirds of the GDM–CTS effect transmitted through CCS-related pathways of oxidative stress and metabolic dysfunction. At the protein level, pQTL-based MR and COLOC analyses implicated CCS, HEXIM2, HEXIM1, CPZ, and PTPN9 as causal CTS-related proteins, underscoring a complex network of protein-level perturbations in disease development. In parallel, eQTL-based transcriptomic analyses identified five immune-related genes—TOB2, ZBTB34, HLA-DRB5, RNF123, and CCS—as colocalised with CTS risk and functionally linked to inflammation, antigen presentation, and fibrotic remodelling. Together, these convergent lines of evidence support oxidative stress, immune dysregulation, and chronic inflammation as core molecular mechanisms linking GDM to CTS. Clinically, they argue for strict glycaemic control, proactive neurological surveillance, and consideration of targeted antioxidant or immune-modulatory strategies in women with GDM, particularly in late pregnancy. This strategy uses MR and multi-omics research to show how gestational diabetes mellitus can lead to carpal tunnel syndrome, find the molecular targets that can be treated with drugs, and explain how we can prevent carpal tunnel syndrome in women who are at risk.

## Data Availability

The original contributions presented in the study are included in the article/**Supplementary Material**. Further inquiries can be directed to the corresponding author.
